# Supplementary material for: The Meq Genes of Nigerian Marek’s Disease Virus (MDV) Field Isolates Contain Mutations Common to Both European and US High Virulence Strains
Source: Viruses. 2024 Dec 31;17(1):56. doi: 10.3390/v17010056 (PMC11769123; doi:10.3390/v17010056)
Supplement: Supplementary file 1 [file viruses-17-00056-s001.zip › viruses-3252745-supplementary.pdf]

viruses

MDPI

Supplementary Materials:

Supplemental Table S1. Molecular analysis of Nigerian MDV DNA samples

Sample ID	gL size[a] (bp)	PCR-RFLP gL[b] genotype (bp)	Meq amplicon size (bp)	Viral genome copy # /10,000 cells	ALV-J	CIAV	REV
CMB-LV	-	-	-	1.95×10^2			
CMB-SP	-	-	1,020	2.9×10^2			
CMB-VB	771	359, 251, 161	1,020	nd[c]			
CMB-FT	771	359, 251, 161	1,020	1.87×10^4	Neg.[d]	**Pos.**[e]	**Pos.**
BRM-LV	771	359, 251, 161	1,020	2.10×10^2	Neg.	Neg.	Neg.
BRM-HB	771	359, 251, 161	1,020	5.00×10^1			
EB1-SP	-	-	1,020	3.80×10^2			
EB1-LV	-	-	1,020	nd			
EB1-LG	771	359, 251, 161	1,020	nd			
EB1-HT	771	359, 251, 161	1,020	1.04×10^3	Neg.	**Pos.**	**Pos.**
EB2-FT	771	359, 251, 161	1,020	1.30×10^1	Neg.	**Pos.**	**Pos.**
EB2-VB	-	-	Nd	1.10×10^1			
EB2-HT	nd	-	-	nd			
NGH-FT	-	-	1,020	3.13×10^3			
NGH-BD	nd	-	1,020	7.60×10^2	Neg.	Neg.	Neg.
LEC-LV	-	-	1,020	5.00			
LEC-HT	771	359, 251, 161	1,020	4.90×10^2			
LEC-SP	-	-	1,020	9.00			
LEC-LG	771	359, 251, 161	1,020	8.10×10^1	Neg.	Neg.	Neg.
MH1-HT	-	-	1,020	nd			
MH1-LV	771	359, 251, 161	1,020	1.10×10^1	Neg.	Neg.	Neg.
MH1-SP	-	-	nd	nd			
MH1-LG	771	359, 251, 161	1,020	3.60×10^1			
MH2-OV	771	359, 251, 161	1,020	4.00×10^1	Neg.	Neg.	Neg.
MH2-KD	771	359, 251, 161	1,020	nd			
MH2-FT	-	-	1,020	1.56×10^4			
WKB-BL	-	-	1,020	nd			
WKB-FT	-	-	1,020	nd			
WKB-LV	nd	-	-	nd	Neg.	Neg.	Neg.
WKB-SP	nd	-	nd	nd			
RT-FT	-	-	1,020	2.07×10^4			
RT-BD	-	-	-	1.10×10^1			
RT-LV	nd	-	nd	1.10×10^1	Neg.	Neg.	Neg.
RT-HT	nd	-	-	nd			
GDA-FT	nd	-	1,020	Nd			

GDA-VB	nd	-	1,020	Nd	Neg.	Neg.	Neg.
BBL-VB	-	-	1,020	Nd	Neg.	Neg.	Neg.
BBL-FT	nd	-	1,020	Nd			
BBL-SP	-	-	nd	Nd			
BBL-LV	nd	-	1,020	Nd			

[a] – observed size of gL PCR product in bp, without deletion, [b] – observed size of expected gL PCR product in bp, without deletion, [c] – nd, not detected, [d] – Neg., negative by endpoint PCR, [e] – Pos., positive by endpoint PCR, †

Supplemental Table S2. Primers used for screening of adventitious agents (ALV-J, CIAV, and REV).

Agent	Primer Sequences	Amplicon Size	Reference
ALV-J	FOR: 5′-AATTCTGCTTGAAATATG-3′	454 bp	This report[1]
	REV: 5′ -AGTTGTCAGGGAATCGAC-3′		
CIAV	FOR: 5′-CTAAGATCTGCAACTGCGGA-3′	420 bp	NVSL[2]
	REV: 5′ -CCTTGGAAGCGGATAGTCAT-3′		
REV	FOR: 5′ -CATACTGGAGCCAATGGTT-3′	292 bp	Rath et al.,
	REV: 5′ -AATGTTGTACCGAAGTACT-3′		2003[3]

[1] – designed using published sequence of HPRS-103 for E (XSR) region

[2] - Taylor SP, National Veterinary Services Laboratory (NVSL), USDA, ARS, Ames, IA.

[3] – from Table 3 in [103]

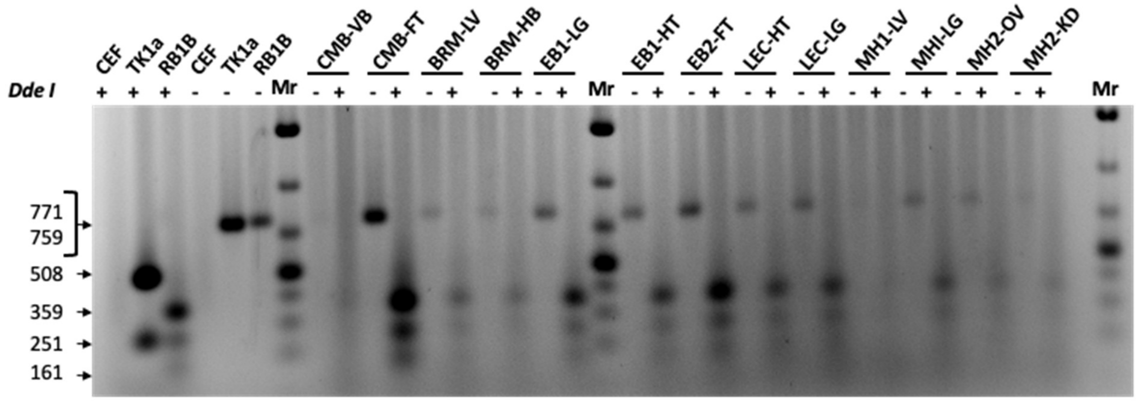

Supplemental Figure S1: PCR/RFLP analysis of the glycoprotein L (gL) locus. The Nigerian field strain gL amplicons were resolved on an agarose gel for the PCR/RFLP detection of a common 12 nt deletion. The gL locus was amplified by PCR to yield a 771 bp or 759 bp amplicon. Amplicons are shown with and without *DdeI* digestion. The gL deletion is characterized by the 759, 508, and 251 bp bands, whereas the 771, 359, 251, and 161 bp bands indicate strains without the deletion.

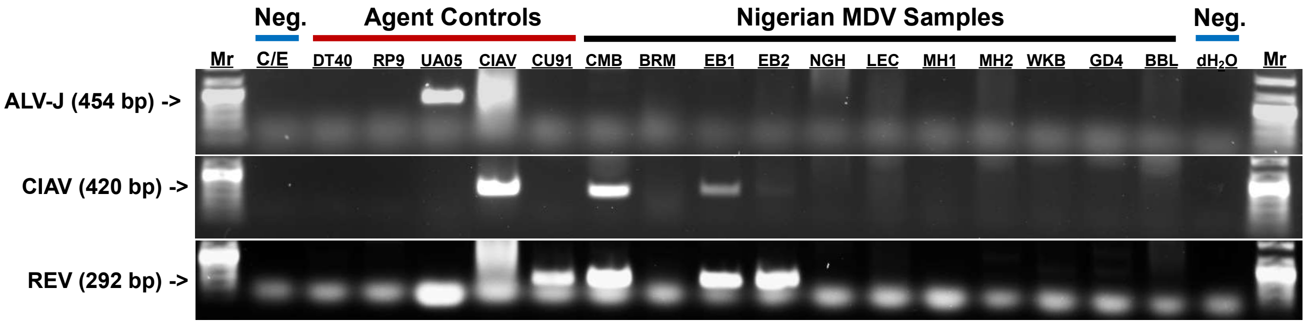

Supplemental Figure S2: PCR analysis for Adventitious Agents: ALV-J, CAV and REV. Select DNA samples from each of the locations were used as templates for PCR analysis for Avian leukosis virus, subgroup J (ALV-J), chicken infectious anemia virus (CIAV) and reticuloendotheliosis virus (REV). Primers are given in Table Supplemental Table 2. The controls used were DNA isolated from line 0 fibroblasts (C/E), ALV, subgroup A (DT40 cell line), ALV, subgroup B (RP9 cell line), ALV, subgroup J (UA05 myeloid cell line, established by MSP in 2000), a commercial MDV isolate from 2005 (MDV and CIAV positive), reticuloendotheliosis virus (REV A/T, CU91 cell line). The Nigerian samples examined are given in the Supplementary Table 1 (denoted with †). PCR conditions were 10 μM of each primer, 25 – 100 ng of each template, and the polymerase used was HiFi 2X Polymerase mix (ThermoFisher) with amplification conditions of 5 min at 94˚C, 35 cycles of 1 min at 94˚C, 1 min at 60˚C and 1 min at 72˚C, followed by final extension of 5 min at 72˚C. Amplicons were separated on a 1.2% agarose gel in 1X TAE buffer and DNA was visualized using SybrSafe dye.

References

103. Rath, N.C.; Parcells, M.S.; Xie, H.; Santin, E. Characterization of a Spontaneously Transformed Chicken Mononuclear Cell Line. *Vet Immunol Immunopathol* 2003, *96*, 93–104, doi:10.1016/S0165-2427(03)00143-0.
